# Autonomic dysfunction in COVID-19 patients receiving mechanical ventilation: A cross-sectional study

**DOI:** 10.1590/1516-3180.2022.0513.R1.09022023

**Published:** 2023-04-17

**Authors:** Renata Baltar da Silva, Victor Ribeiro Neves, Mayara Costa Barros, Bruno Bavaresco Gambassi, Paulo Adriano Schwingel, Dário Celestino Sobral

**Affiliations:** I PT, MSc. Physiotherapist and Doctoral Student, Programa de Pós-Graduação em Ciências da Saúde (PPGCS), Universidade de Pernambuco (UPE), Recife (PE), Brazil. Physiotherapist, Hospital das Clínicas of the Universidade Federal de Pernambuco (HC-UFPE), Empresa Brasileira de Serviços Hospitalares (EBSERH), Recife (PE), Brazil. Physiotherapist, Agamenon Magalhães Hospital (HAM), UPE, Recife (PE), Brazil.; Hospital das Clínicas of the Universidade Federal de Pernambuco, Empresa Brasileira de Serviços Hospitalares, Recife, PE, Brazil; Agamenon Magalhães Hospital, Recife, PE, Brazil; II PT, PhD. Physiotherapist and Adjunct Professor, Programa de Pós-Graduação em Reabilitação e Desempenho Funcional (PPGRDF), Universidade de Pernambuco (UPE), Petrolina (PE), Brazil.; III PT. Physiotherapist and Master's Student, Programa de Pós-Graduação em Saúde Translacional (PPGST), Universidade Federal de Pernambuco (UFPE), Recife (PE), Brazil.; IV PhD. Physical Educator and Adjunct Professor, Programa de Pós-Graduação em Gestão de Programas e Serviços de Saúde (MGPSS), Universidade Ceuma (UniCEUMA), São Luís (MA), Brazil.; V PhD. Sports Physiologist and Associate Professor, Programa de Pós-Graduação em Ciências da Saúde (PPGCS), Universidade de Pernambuco (UPE), Recife (PE), Brazil.; VI MD, PhD. Physician and Associate Professor, Programa de Pós-Graduação em Ciências da Saúde (PPGCS), Universidade de Pernambuco (UPE), Recife (PE), Brazil.

**Keywords:** Autonomic nervous system, Heart rate, SARS-CoV-2, Critical care, Heart rate variability, Intensive care unit, Mechanical ventilatory assistance

## Abstract

**BACKGROUND:**

Coronavirus disease 2019 (COVID-19) can damage cardiac tissue by increasing troponin levels and inducing arrhythmias, myocarditis, and acute coronary syndrome.

**OBJECTIVES:**

To analyze the impact of COVID-19 on cardiac autonomic control in mechanically ventilated intensive care unit (ICU) patients.

**DESIGN AND SETTING:**

This cross-sectional analytical study of ICU patients of both sexes receiving mechanical ventilation was conducted in a tertiary hospital.

**METHODS:**

Patients were divided into COVID-19-positive (COVID(+)) and COVID-19-negative (COVID(-)) groups. Clinical data were collected and heart rate variability (HRV) records obtained using a heart rate monitor.

**RESULTS:**

The study sample comprised 82 subjects: 36 (44%) in the COVID(-) group (58.3% female; median age, 64.5 years) and 46 (56%) in the COVID(+) group (39.1% females; median age, 57.5 years). The HRV indices were lower than the reference values. An intergroup comparison identified no statistically significant differences in the mean normal-to-normal (NN) interval, standard deviation of the NN interval, or root mean square of successive differences in NN intervals. The COVID(+) group had an increased low frequency (P = 0.05), reduced high frequency (P = 0.045), and increased low frequency/high frequency (LF/HF) ratio (P = 0.048). There was a weak positive correlation between LF/HF and length of stay in the COVID(+) group.

**CONCLUSION:**

Patients who received mechanical ventilation had lower overall HRV indices. COVID(+) patients who received mechanical ventilation had lower vagal HRV components. These findings likely indicate clinical applicability, as autonomic control impairments are associated with a greater risk of cardiac death.

## INTRODUCTION

Breathing difficulties with severe hypoxemia, caused by infection with the severe acute respiratory syndrome (SARS) virus, is the most important manifestation of coronavirus disease 2019 (COVID-19).^1^ In addition to the possibility of a pulmonary lesion, COVID-19 may directly cause heart damage in the form of myocarditis, heart failure, cardiogenic shock, acute coronary syndrome, and cardiac arrhythmias. Clinical manifestations are also accompanied by increased cardiac biomarker levels. The mechanisms that cause these cardiovascular disorders are not yet clear; however, they are believed to be related to excessive inflammatory responses, hypoxemia, thromboembolic phenomena, and endothelial dysfunction.^2,3^ The severity of infection increases simultaneously with the activation of the inflammatory pathways that trigger cytokine storm.^4^

Cardiac autonomic control can be studied through heart rate variability (HRV), which is the physiological phenomenon of variation in the time interval between heartbeats.^5,6^ Decreased HRV is a sign of abnormal and insufficient adaptation of the autonomic nervous system (ANS) and may indicate physiological malfunctioning in some clinical conditions.^7–10^ Autonomic dysfunction is common in various disorders that occur in patients with critical conditions, such as multiple organ dysfunction syndrome, sepsis, myocardial infarction, decompensated heart failure, and severe brain injury.^11–14^

Furthermore, depressed parasympathetic activity has been implicated in the pathogenesis of diseases associated with excessive inflammatory responses.^15^ These changes may be clarified by the inflammatory reflex theory – i.e., activation of the vagus nerve and consequently reduced inflammatory responses in septic and aseptic inflammation models.^16^ Reduced HRV may be an independent predictive factor of 30-day all-cause mortality in intensive care unit (ICU) patients.^15,17–19^

## OBJECTIVE

Given the likely cardiac damage caused by COVID-19, the objective of this study was to analyze the impact of this disease on cardiac autonomic control in ICU patients.

## METHODS

### Study design

This cross-sectional analytical study was conducted between August 2020 and February 2021 in an ICU that exclusively treated adult SARS patients. The following data were collected from the medical records: sample characterization (such as sex, age, vital signs (heart rate [HR], systolic arterial pressure, diastolic arterial pressure, peripheral oxygen saturation [SpO_2_]), history of current disease, pre-existing diseases, and ICU length of stay).

Additionally, ventilatory parameters (positive end-expiratory pressure, pressure support [PS], fraction of inspired oxygen [FiO_2_], and arterial pressure of oxygen/FiO_2_ ratio [PaO_2_/FiO_2_]) and ventilatory muscle function data (maximum inspiratory pressure, maximum expiratory pressure, and rapid and shallow breathing index) were collected.

The samples were obtained for convenience. Participants in the study included patients receiving mechanical ventilation (MV) who underwent reverse transcription-polymerase chain reaction (RT-PCR) for COVID-19 diagnosis. Those with positive and negative results were included, and their groups were denoted COVID(+) and COVID(−), respectively. Patients with complex arrhythmias, second- or third-degree atrioventricular block, cardiac pacemakers, heart transplants, or those taking antiarrhythmic drugs were excluded.

This study was approved by the Research Ethics Committee of the HUOC/PROCAPE Hospital Complex (no. CAAE 13364019.5.0000.5192) on June 26, 2019. The patients or their legal guardians signed an informed consent form.

### Heart rate variability

HRV was measured with a Polar V800 heart rate monitor (Polar Electro Oy, Kempele, Finland), with a Polar H10 heart rate sensor (Polar Electro Oy) positioned at the patient's xiphoid process with a Polar Pro strap (Polar Electro Oy). The final data were exported to Kubios HRV Standard software (release 3.3.1, 2019; Kubios Oy, Kuopio, Finland), in which normal-to-normal intervals (NNi) were processed and digitally filtered to eliminate artifacts. The Task Force of the European Society of Cardiology and North American Society of Pacing and Electrophysiology recommendations were followed.^20^

All patients received invasive pressure support MV and were always assessed in the morning, between 8 o'clock and noon, to avoid the influence of the circadian rhythm. The subjects were lying in bed in the supine position, with the headrest angled at 45°. Time and frequency domains were analyzed with the highest quality and fewest-artifact 5-minute extracts.

The following HRV data were investigated in the time domain: mean NNi (ms), standard deviation of the NN interval (SDNN, ms), and the root mean square of successive differences in NN intervals (RMSSD, ms). For the frequency domain, low frequency (LF, in normalized units [nu]), high frequency (HF, nu), and the LF/HF ratio were analyzed.

### Statistical analysis

The statistical analysis was performed using SPSS software (release 22.0, 2013; SPSS Inc., Chicago, Illinois, United States, Release 22.0, 2013). Initially, normality was verified using the Kolmogorov-Smirnov test and homoscedasticity using Bartlett's test. Given the results and considering the nature of the study, continuous variables were presented as medians (first quartile – third quartile) (minimum value – maximum value) and categorical variables as absolute and relative frequencies. The Mann-Whitney U test compared the results of the continuous variables between the two groups, while the Pearson chi-square test (χ^2^) analyzed the proportions of categorical variables. The measures of central tendency and dispersion presented in the study by Nunan were taken as normal reference values of the HRV parameters analyzed in the present study.^21^ A linear regression was performed to evaluate possible confounding factors. All analyses were bilateral and performed at the 5% significance level. When calculated, P values and 95% confidence intervals were precise.

## RESULTS

The study comprised 82 individuals divided into two groups based on RT-PCR results for SARS-CoV-2. The COVID(-) group had 36 (44%) subjects with a median age of 64.5 (56.0–70.0) years; 21 (58.3%) were female. The COVID(+) group had 46 (56%) subjects with a median age of 57.5 (42.8–73.0) years; 18 (39.1%) were female. The groups were homogeneous, and the sample characterization data are presented in Table 1.

**Table 1:** under mechanical ventilation (n = 82)

Variables	Negative COVID (n = 36)	Positive COVID (n = 46)	P
	Median (1Q–3Q) [Min-Max]	Median (1Q–3Q) [Min-Max]	
Age, years	64.5 (56.0–70.0)[28.0–81.0]	57.5 (42.8–73.0)[31.0–88.0]	0.472
Heart rate, bpm	93.0 (81.3–101.0)[58.0–119.0]	88.0 (73.0–104.0)[55.0–147.0]	0.492
Systolic blood pressure, mmHg	137.0 (113.8–156.5)[67.0 - 189.0]	128.5 (108.8–142.5)[64.0–166.0]	0.071
Diastolic blood pressure, mmHg	74.5 (66.3–86.8)[47.0–100.0]	69.5 (60.0–84.0)[48.0–117.0]	0.184
Peripheral capillary oxygen saturation, %	97.0 (95.0–98.0)[93.0–100.0]	95.5 (94.0–98.0)[91.0–100.0]	0.035
Length in intensive care unit, days	5.0 (3.0–10.8)[1.0–26.0]	9.0 (4.0–14.0)[1.0–34.0]	0.024
Positive end-expiratory pressure, cmH^2^O	6 (6–8)[5–10]	6 (6–8)[5–10]	0.795
Pressure support, cmH^2^O	10 (10–10)[8–18]	10 (10–12)[8–16]	0.682
Fraction of inspired oxygen, %	21 (21–30)[21–35]	25 (21–35)[21–50]	0.024
PaO^2^/FiO^2^ ratio	366 (310–471)[201–666]	356 (277–419)[140–633]	0.168
Maximal inspiratory pressure, cmH^2^O	-60 (-47 – -80)[-20 – -120]	-60 (-60 – -100)[-30 – -120]	0.282
Maximal expiratory pressure, cmH^2^O	80 (46–100)[20 – 170]	70 (48–100)[20–150]	0.828
Rapid shallow breath index	44 (36–66)[12–110]	46 (37–66)[24–85]	0.602
Female sex, n (%)	21 (58.3%)	18 (39.1%)	0.084
Systemic arterial hypertension, n (%)	24 (66.7%)	24 (52.2%)	0.186
Diabetes mellitus, n (%)	19 (52.8%)	18 (39.1%)	0.218
Chronic obstructive pulmonary disease, n (%)	6 (16.7%)	5 (10.9%)	0.523

COVID-19 = coronavirus disease 2019; 1Q = first quartile; 3Q = third quartile; Min-Max = minimum-maximum; PaO^2^ = oxygen blood pressure; FiO2 = fraction of inspired oxygen.

No difference in vital signs was observed between the two groups, except for SpO_2_, which was significantly lower in the COVID(+) than COVID(-) group (95.5% versus 97.0%; P = 0.035). Nonetheless, both values were normal. Among the ventilatory parameters, the FiO_2_ used was higher in the COVID(+) group (P = 0.024). There was no significant intergroup difference in ventilatory muscle function.

The main comorbidities found in the COVID(+) group were systemic arterial hypertension (52.2%) and diabetes mellitus (39.1%), with a statistically similar prevalence, in contrast to the COVID(-) group. The patients' length of stay by the day of assessment was significantly longer in the COVID(+) group (P = 0.024) than in the negative group (Table 1).

All HRV parameter values for the patients in both groups were significantly lower than the reference values. In contrast, the comparison of time domain indicators between the COVID(+) and COVID(-) groups revealed no statistical difference in mean NNi, SDNN, or RMSSD values. In the frequency domain, comparison between the groups revealed a significant increase(P = 0.05) in LF, a significant decrease (P = 0.045) in HF, and an increase in the LF/HF ratio (P = 0.048) in the COVID(+) group (Table 2).

**Table 2:** Measures of heart rate variability of positive and negative patients for COVID-19, admitted to the intensive care unit under mechanical ventilation (n = 82)

Variables	Negative COVID (n = 36)	Positive COVID (n = 46)	P
	Median (1Q–3Q) [Min-Max]	Median (1Q–3Q) [Min-Max]	
NNi medium, ms	639.5 (572.3–764.8)[451.0–1191.0]	653.0 (571.8–800.8)[8.6–1321.0]	0.581
SDNN, ms	8.1 (4.5–25.9)[3.0–65.0]	10.4 (6.2–21.3)[2.5–46.3]	0.562
RMSSD, ms	12.1 (4.5–32.2)[2.1–98.0]	12.9 (7.9–27.3)[2.0–68.6]	0.695
LF, nu	33.6 (24.8–55.4)[9.2–87.2]	47.2 (26.4–71.0)[7.2–87.8]	0.050
HF, nu	66.8 (44.3–74.9)[12.8–90.2]	51.4 (28.9–71.8)[12.2–91.4]	0.045
LF/HF ratio	0.5 (0.3–1.3)[0.1–6.8]	0.9 (0.4–2.5)[0.1–7.2]	0.048

COVID-19 = coronavirus disease 2019; 1Q = first quartile; 3Q = third quartile; Min-Max = minimum-maximum; HF = high frequency; LF = low frequency; NNi = N-N interval; RMSDD = square root of the mean squared differences of successive N-N intervals; SDNN = standard deviation of the N-N interval.

**Figure 1:**
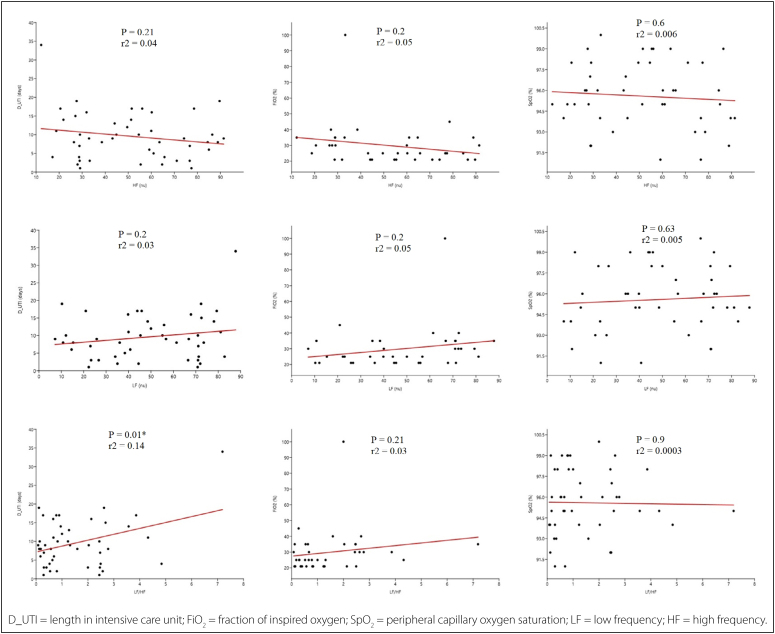
Linear regression analysis between indices in the frequency domain and possible confounding factors days of ICU stay, SpO_2_, and Fio2.

The indices in the frequency domain that showed a significant difference in the COVID(+) group were subjected to linear regression to analyze possible confounding factors, and a weak positive correlation was observed between LF/HF and days spent in the ICU (P = 0.01; r2 = 0.14) (Figure 1).

## DISCUSSION

This study observed changes in cardiac autonomic control interactions in ICU patients receiving invasive MV, whose RT-PCR test was positive for COVID-19. These patients had lower vagal activity and sympathetic hyperactivity in the frequency domain than non-infected patients.

Strong hyperimmune reactions due to COVID-19 produce a large adrenergic release, which is mainly modulated by the sympathetic nervous system.^2,3^ Consequently, a modulated compensatory response occurs via the cholinergic anti-inflammatory pathway of the parasympathetic nervous system.^4,5^ Thus, the ANS participates in regulating this inflammatory reflex, and its balance is essential for maintaining physiological homeostasis.^11,12^

The vagus nerve is an important neuroimmunomodulator of the anti-inflammatory pathway.^22^ When working properly, this regulatory anti-inflammatory response limits viral infection dissemination and is vital for controlling and treating COVID-19. However, when vagal activity is reduced, the inflammatory response may get out of control, contributing to hyperinflammation – the so-called cytokine storm.^23^ In this regard, the results of this research show decreased HF in the COVID(+) group, demonstrating that these patients' vagal component is reduced. Hence, unregulated immune responses observed in severe cases of COVID-19 (those which cause inflammation and SARS) may result from impaired vagal activity in inflammation regulation.^22^

Various previous studies have already researched the correlation between HRV and inflammatory markers.^24–26^ Tateishi et al. found that interleukin-6 (IL-6) was negatively correlated with LF in septic ICU patients.^24^ Papaioannou et al. reported an inverse correlation between LF and LF/HF and C-reactive protein levels.^25^

Previous studies examining the role of HRV in COVID-19 found that a reduction in HRV preceded an increase in inflammatory markers. However, these studies used small sample sizes and did not statistically adjust for important confounders such as age and comorbidities.^27,28^

One of the first studies examining the potential role of HRV as a surrogate marker for vagus nerve activity in COVID-19 showed that age is a predictor of death only in cases of reduced HRV. This suggests that the vagus nerve plays an important moderating and protective role in COVID-19 and may even weaken the prognostic role of aging.^27^

Among the HRV parameters analyzed in the time domain, SDNN and RMSSD are markers of parasympathetic tone. Their values were low in both groups in the present study, demonstrating that parasympathetic activity was reduced in patients with severe disease who were receiving MV. This reduction was sharper in the COVID(-) group, although the difference was not statistically significant. A cross-sectional analytical study conducted in India also found significantly higher RMSSD and SDNN values in the COVID(+) group.^29^ However, that study neither included severe patients nor used oxygen therapy.

Jarczok et al. observed in a cross-sectional study that daytime RMSSD values below 25 (±4) ms indicate high cardiovascular risk.^30^ Hence, the low RMSSD values found in this study suggest that ICU patients, with or without a confirmed COVID-19 diagnosis, were at an increased cardiovascular risk.

HRV reductions have been associated with disease severity and increased mortality in ICU patients.^11^ Papaioannou et al. observed that less clinically stable patients have a lower LF/HF ratio and decreased overall variance; they also pointed out that patients recovered from such reduction as they improved and were discharged from the ICU.^25^ Likewise, LF/HF values in the present study were lower in both groups, demonstrating that the sample patients were in a severe condition.

The average length of stay for the COVID(+) group was 9 days. It is known that in the first two weeks of infection, the defense mechanisms are deregulated and the severity of the disease increases as the cytokine storm is activated.^4,31^ A retrospective study conducted in China analyzed chest computed tomography (CT) scans of 121 patients with COVID-19 and showed the most extensive disease approximately 10 days after the onset of symptoms.^32^ A study in Mexico of COVID-19 patients observed that the interval between the first symptoms and death was a mean 9 (range, 5–13) days.^33^ Thus, the weak positive correlation between LF/HF and days spent in the ICU observed in the COVID(+) group may be related to the greater dysregulation of the anti-inflammatory reflex observed in the initial 10 days of the disease.

### Limitations

This study has some limitations. The pandemic period and difficulty in obtaining an interruption-free HRV record due to electronic equipment causing interference in the ICU environment compromised the recruitment of a larger sample, which may have limited the generalization of our findings. Recent studies have shown that short-term recordings of HRV indices in the time domain may not be monitored to interpret oscillations in autonomic and regulatory nervous systems. This may explain the lack of significant differences in the RMSSD and SDNN between the groups in the present study.

Although HRV spectral analysis is an accepted, valid, and reliable noninvasive indicator of ANS balance, no other measures (such as catecholamine serum levels or baroreflex sensitivity) were used to collect data on autonomic activity.

## CONCLUSIONS

ICU patients who received MV had lower overall HRV measures. HF reduction was particularly sharper in COVID-19 patients receiving MV, which demonstrates the role of cardiac autonomic control in the pathogenesis of diseases characterized by excessive inflammatory responses. Hence, HRV measurements with spectral analysis can be promising markers of the inflammatory response, aiding future studies on new anti-inflammatory treatments. The findings of the present study are likely to be clinically applicable, as autonomic control impairments are associated with a greater risk of cardiac death. Further studies are required to confirm these results.
